# Laryngeal Cancer in Women: Unveiling Gender-Specific Risk Factors, Treatment Challenges, and Survival Disparities

**DOI:** 10.3390/curroncol32010019

**Published:** 2024-12-29

**Authors:** Barbara Verro, Simona Fiumara, Giuseppe Saraniti, Carmelo Saraniti

**Affiliations:** 1 Division of Otorhinolaryngology, Department of Biomedicine, Neuroscience and Advanced Diagnostic, University of Palermo, 90127 Palermo, Italy; carmelo.saraniti@unipa.it; 2 Speech Therapist, Private Practice, 90100 Palermo, Italy; simona.fiumara89@gmail.com; 3 Ospedale Buccheri La Ferla, 90123 Palermo, Italy

**Keywords:** laryngeal cancer, woman, gender discrepancies, hormonal factors

## Abstract

Laryngeal cancer is one of the main causes of morbidity and mortality worldwide, with a significantly higher prevalence among men than women. However, the incidence, clinical characteristics, and specific treatment of laryngeal cancer in women have often been overlooked by research. This review aims to examine gender differences in incidence, risk factors, hormonal mechanisms, survival, and therapeutic approaches for laryngeal cancer in women. Although smoking and alcohol remain the main risk factors, evidence suggests that women may be more vulnerable to the harmful effects of these behaviors, with a relative higher risk than men. In addition, hormonal factors such as estrogen may influence women’s susceptibility to laryngeal cancer, accelerating tumor growth and complicating treatment. Differences in treatment between the sexes, with women tending to receive less intensive treatment than men, is another crucial aspect that needs more attention. This article also analyses the disparities in survival, highlighting that women often have a better prognosis, although this trend varies according to demographic characteristics and the health system. The increasing incidence of laryngeal cancer in women requires increased research to fully understand risk factors and underlying biological mechanisms in order to develop more personalized treatments and optimize clinical outcomes for patients.

## 1. Introduction

Laryngeal cancer (LC) is a major cause of morbidity and mortality, with approximately 12,000 new cases and nearly 4000 deaths annually in the United States alone [1,2]. The 2022 Global Cancer Observatory (GLOBOCAN) reported 189,191 new cases and 103,359 deaths per year in the world [3]. The incidence of LC is much higher in men than in women, with a male-to-female (M:F) ratio of approximately 4:1 [4,5]. This ratio is highest in people over 60 years old [6]. The female age-standardized incidence rate in the world per 100,000 persons is higher in Cuba (2.3), followed by in Brazil, the Syrian Arab Republic, the Dominican Republic, Bangladesh, Iran, Iraq, Azerbaijan, Poland, Hungary, and Ireland (0.9–1.4) [7]. In contrast, the lowest incidence rates have been observed in Benin and Guadeloupe (France), with much lower values of 0.07 and 0.09 cases per 100,000 inhabitants, respectively. This suggests significant geographical variability in the spread of the disease, with important differences between different countries and regions [8]. Over the years, the overall incidence of LC has decreased in both sexes, though the reduction has been more significant in men, likely due to a decline in smoking and alcohol consumption. The reduction among women has been less pronounced, which could be due to the increasing prevalence of smoking and alcohol use among women in past generations [9]. However, after 35 years, all indicators are steadily increasing. The incidence of LC peaks after 65 years in both men and women, then stabilizing at about 4 new cases per 100,000 women and about 25–30 new cases per 100,000 men [10]. The prevalence of LC shows a peak between 60 and 80 years, recording 17–20 cases per 100,000 women and 120–140 cases per 100,000 men, then gradually decreasing. The mortality rate reflects the trend in incidence, peaking at around 80 years for both sexes and remaining relatively stable with 23 deaths per 100,000 men and 3 deaths per 100,000 women [11,12]. Despite this, research focused specifically on LC in women remains limited, which may be contributing to the poorer understanding of gender differences in the disease. One of the reasons for this disparity could be the historical view of the larynx as a secondary sex organ, as it is affected by sex hormones during puberty and throughout the menstrual cycle. This means that the larynx undergoes changes related to sex hormones, such as estrogen and androgens, which affect the development and functioning of this area of the body. However, recent studies suggest that hormones, including estrogen and androgens, could play a significant role in the development and progression of LC [13].

This review explores the incidence, risk factors, hormonal influences, and treatment outcomes of LC in women, highlighting key differences from male patients.

## 2. Search Methodology and Data Analysis

The bibliographical research was carried out in the main scientific databases PubMed (https://pubmed.ncbi.nlm.nih.gov accessed on 30 November 2024) and Scopus (https://www.scopus.com accessed on 30 November 2024), running the following keywords strings: *laryngeal cancer* OR *laryngeal carcinoma* AND *women* OR *female* OR *sex* OR *gender*.

Inclusion criteria were studies about laryngeal cancer, any histological type of laryngeal carcinoma, articles published after 2000, both retrospective and prospective studies, and articles written in the English and/or Italian language.

Exclusion criteria were case reports; studies about benign laryngeal lesions; studies that did not distinguish the results according to the sex of patients; and studies assessing head and neck cancers overall, without distinction by anatomical region.

**Figure 1:**
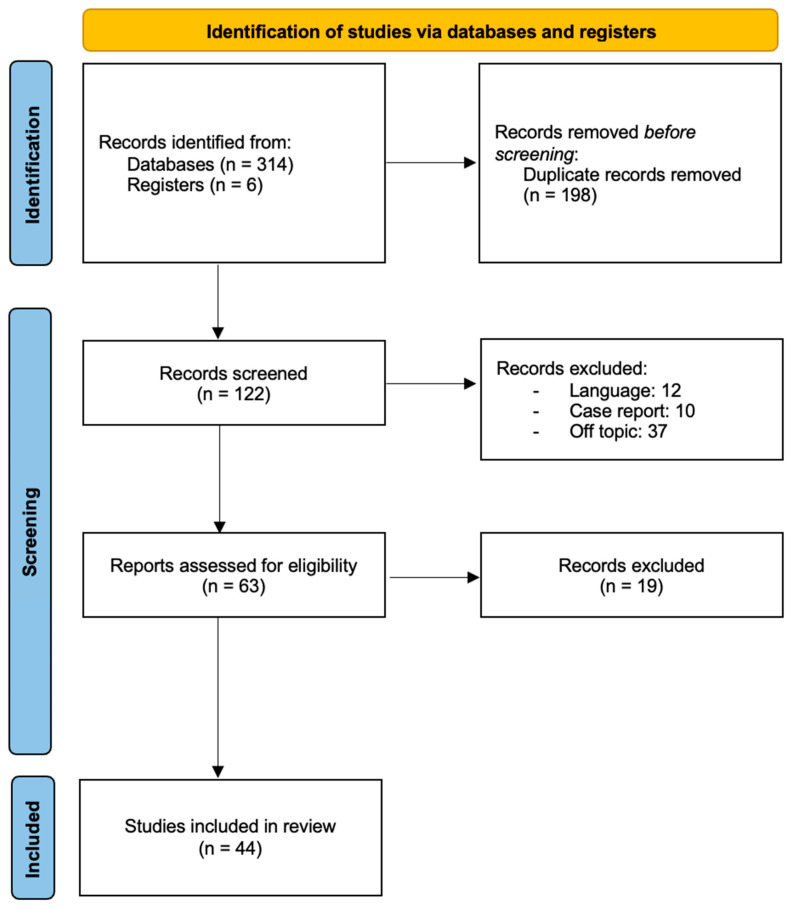
PRISMA 2020 flow diagram of study selection process of the literature.

In conclusion, 314 articles were found from the database searching and 6 articles were identified from the references of selected articles. After removing duplicates (198), two authors (B.V. and C.S.) screened the articles by reading titles and abstracts (122 articles) according to the selection criteria: language (12), case reports (10), off topic (37). Secondly, the 63 selected full-text manuscripts were read by the same two authors including this review’s 44 articles (Figure 1) [14].

## 3. Main Risk Factors

As known, smoking and alcohol consumption represent the main risk factors for LC in both sexes. However, additional factors may be involved in LC occurrence in women. Data from the 2020 Global Cancer Observatory (GLOBOCAN) [15] showed that the increased risk of LC in women was correlated to Human Development Index (*p*-value < 0.001), obesity (*p*-value 0.004), hypertension (*p*-value 0.024), inactivity (*p*-value 0.011), diabetes (*p*-value < 0.001), smoking (*p*-value < 0.001), and lipid disorders (*p*-value < 0.001). Female mortality results were related to alcohol abuse (*p*-value < 0.001) and lower gross domestic product per capita (*p*-value 0.003) [16]. Gallus et al. carried out a study on 68 Italian women affected by LC, finding that tobacco smoking represents the main risk factor as in men, with a higher relative risk for women (odds ratio OR = 435.7). Alcohol consumption showed a significant role in LC occurrence only in the case of heavy drinkers: it is a less important risk factor than smoking (OR = 4.3) [17]. A study with 795,121 female participants confirmed that drinking alcohol increases the risk of LC (relative risk RR = 1.35), most of all in heavy smokers (RR = 9.70), confirming the hypothesis that alcohol exacerbates the negative effects of smoking [18].

In addition to behavioral factors, the role of hormones in LC occurrence has been suggested over the years. In 1989, Yang et al. hypothesized that the different sex incidence of LC might be related to different laryngeal anatomy. In particular, the author found a higher M:F ratio in the case of glottic cancer assuming that this result could also be explained by the different characteristics of vocal cords: men have thicker and longer vocal cords and use a lower pitch tone. Maybe, these features may lead to valid (or not) mucosal clearance of inhaled carcinogens [19]. On the contrary, other researchers found that the supraglottic region is the most affected by laryngeal cancer in females (ranging from 24.4% to 61.2% of cases, based on studies) [4,20].

Environmental factors, including occupational exposures, may also play a role in LC risk. The ICARE study, conducted on 296 French female patients affected by head and neck cancer, including LC (47 patients), found a correlation between blacksmithing and LC and the absence of an association with char workers, cleaners, and related workers, despite exposure to solvents, chemical agents, and dust [21]. Asbestos is another occupational risk factor, classified ad “carcinogenic to humans” by the IARC. Studies demonstrated its correlation to laryngeal cancer, with an RR of 1.4 [22,23]. However, a statistically significant correlation between asbestos exposure and LC was found in men (*p*-value 0.001), but not in women (*p*-value 0.125). The authors hypothesized that the small sample of female patients and the lower incidence of smoking do not allow us to achieve statically significant results [24,25]. A correlation between LC in women and electronic and electric equipment assemblers have shown controversial results: there is a higher LC risk in few studies [21,26], yet no risk in others [27]. These findings suggest that environmental and workplace exposures should also be considered when assessing risk, particularly for women in specific occupations.

## 4. Hormonal Factors

Hormonal, reproductive, and menstrual factors were evaluated to establish their role in LC in women. However, Gallus et al. [17] did not find any relation between age at menarche, artificial menopause, abortions, age at menopause, number of births, oral contraceptive use, hormone replacement therapy, and the risk of LC. On the contrary, Hashim et al. demonstrated that early menopause (before 52 years old) is related to a higher risk of head and neck cancer (OR 1.69 before 52 years vs. 1.59 after 52 years), maybe due to the loss of the protective role of estrogen [28].

Also, LC during pregnancy was evaluated through four cases of laryngeal squamous cell carcinoma (LSCC) and six rare histologies of LC. With regard to LSCC, a diagnosis was made after six months of gestation with healthy babies and an absence of recurrence at 1 year of follow-up. The rare laryngeal tumors were neuroendocrine (death), adenoid cystic, desmoid, myo-epithelial, and paraganglioma [29]. The role of estrogen in desmoid cancer is well known and described in several studies: it has high prevalence during pregnancy, a spontaneous disappearance with menopause, and there is a therapeutic effect of antiestrogen [30].

Placental growth factor (PGF) is a molecule that belongs to the vascular endothelial growth factor (VEGF) family and plays a crucial role in angiogenesis and vasculogenesis. During pregnancy, it is produced mainly by the trophoblast cells, but it is also expressed in other tissues and may be involved in the development of tumors. In the case of LC, it has been observed that cancer cells can secrete PGF, which, once released, stimulates macrophages to change their behavior, a process known as polarization. This change in macrophages leads to the release of a protein called MMP9 (extracellular matrix metalloprotease 9), which is involved in the destruction of the extracellular matrix, a network of proteins that supports cells and tissues. The degradation of the extracellular matrix allows the formation of new blood vessels (neovascularization), essential for growing tumors that need a greater supply of oxygen and nutrients. This process takes place through a cellular signaling pathway called the ERK-MAPK pathway, which is involved in regulating cell growth and survival. So, PGF contributes to the progression of a tumor by promoting the formation of new blood vessels and facilitating the growth of the tumor itself [30].

LSCC appears to show higher expression of androgen, estrogen, and prolactin receptors than that of surrounding normal tissues. This means that cancer cells in the larynx are more sensitive to these hormones, which could affect their behavior. When estrogen (E2) binds to the estrogen α 36 (ERα 36) receptors in LC cells, cell proliferation is stimulated, which means that the tumor cells grow more rapidly. In addition, this bond helps cancer cells to defend themselves against apoptosis induced by chemotherapy. In particular, the rapid activation of enzymes such as protein kinase C (PKC) and phospholipase D is involved in this protection mechanism. In response to the activation of the ERα 36 receptor by estrogen, there is also an increased expression of factors that promote angiogenesis and metastasis. This suggests that the interaction between estrogen and specific receptors in LC cells not only stimulates the growth of the tumor but may also promote its spread and the creation of a vascular network supporting the tumor itself [31,32,33,34].

For the first time, Fei et al. reported that in laryngeal cancer there is a co-expression of prolactin receptors (PRLRs) and estrogen [31]. This means that the presence of these receptors, which are influenced by sex hormones such as prolactin and estrogen, may contribute to the growth and progression of the tumor. The expression of PRLRs has been associated with a worse prognosis, indicating that a tumor may be more aggressive and less sensitive to treatments. In other words, the co-expression of these receptors could make laryngeal cancer more difficult to treat and negatively affect patients’ chances of survival.

## 5. Treatment

Gender differences are also evident in the treatment of LC. Shaikh et al. found that women may be more likely to experience delays in diagnosis and treatment than men. Factors contributing to this delay include socio-economic barriers, differences in symptoms and their perception, as well as possible differences in how women access and interact with the health system [35]. Keirns et al. suggested that this diagnostic delay may be due to reduced clinical suspicion and a lack of screening in women due to the tendency of the disease to affect men more frequently [36]. On the contrary, Majszyk et al. did not find a statistically significant difference in the timing of diagnosis with a shorter (about 3 days) time leading to the first visit and time leading to diagnosis in female patients than in male patients [37].

A 2019 study analyzed the different treatment strategies between men and women affected by head and neck cancer. The authors found that women were undertreated compared to men. They reported a reduced tendency to administer cisplatin in combination with radiotherapy (female vs. male = 34% vs. 44%) as well as to perform radiotherapy (female vs. male = 60% vs. 70%) [38]. On the contrary, Li et al. described a similar therapeutic approach for both sexes in the case of T1 and T3 LC. Women were more likely to undergo radiation therapy than open surgery, most of all in the case of T4 disease [4]. A recent retrospective study found that in the treatment of LC, women were treated more frequently with chemotherapy, often used as a radiosensitizer within chemo-radiotherapy (CRT). This may be because women tend to have a more advanced stage of LC and prefer solutions that preserve the larynx. The higher rate of chemotherapy in women could also be explained by the higher prevalence of supraglottic cancers in this population. However, the multivariate Cox model analysis of the study, which considered both the mode of treatment and the location of the tumor, found no association between these factors and a worsening of DSS, OS, or disease-free survival (DFS) [39]. Similar results were reported by Saini et al. who found that women are less likely to choose a surgical option than men in the case of advanced LC. This may reflect personal preferences, social factors, or differences in perception related to the treatment. In addition, non-surgical treatments such as CRT may be preferred by women to preserve the larynx, since surgery can have significant implications for quality of life with regard to voice and personal image. The study highlights how treatment choices can be influenced by socio-demographic factors and that women may have specific needs and preferences that require a tailored therapeutic approach [40].

A 2021 study carried out on a cohort of patients with head and neck cancer, including LC, from the National Cancer Database showed gender differences in treatment. Indeed, women were more likely to be treated at a university hospital with less cases of private insurance than men (38.0% vs. 40.1%) [41]. An analysis using the National Cancer Database in the United States found that women tend to have more positive margins after transoral laser microsurgery. This may suggest differences related to tumor biology or other factors that influence surgical outcome [42].

These findings emphasize the need for more equitable treatment approaches, ensuring that women with LC receive appropriate care regardless of their gender.

## 6. Survival and Prognosis

Several studies found that women with LSCC have higher overall survival (OS) in comparison with that of men (hazard ratio HR 0.88) [4,43,44]. Li et al. enrolled 17,125 patients affected by LSCC from the Surveillance, Epidemiology, and End Results (SEER) database and found that OS and disease-specific survival (DSS) are higher in females for all stages of LSCC and in the case of glottic and supraglottic cancers. They suggested that this favorable prognosis is related to less exposure to smoking, less tobacco use (packages per year), and a greater tendency to quit smoking after LC diagnosis [4,45]. However, a study carried out on LC women from England and Wales showed different results: women were older and had worse 5-year OS and more advanced LC at diagnosis [20]. Li et al. reported instead that their population is characterized by younger female patients with early LC and better OS. The authors hypothesized that this discrepancy in results could be explained by a different health care system and different characteristics of the populations, with a similar incidence of LC [4]. Wang et al. also have analyzed the differences in survival rates of patients with LSCC between women and men. They have demonstrated that women show better survival rates than men at 1, 3, and 5 years, both for DSS and for OS. For instance, the rate of DSS at 5 years was 65% for women and 59% for men, and the OS at 5 years was 50% for women compared to 44% for men. The authors also found that female sex is a favorable prognostic factor; however, this protection was not observed in patients with subglottic cancer, undifferentiated LC, and in black people [46]. Moreover, a study based on data from the head and neck cancer epidemiology consortium reported a lower risk of LC recurrence in women than in men (HR 0.39) [47]. On the contrary, in multivariate analysis, Haapaniemi et al. reported that female sex represents an independent predictor of high risk of regional recurrence (*p*-value 0.061) [48].

Among men, LC is more aggressive when diagnosed at a younger age, whereas the peak incidence in women occurs between the ages of 40 and 60 [4], although the explanation is not yet known.

Another study focused both on the influence of gender and race in LC survival, finding that black females had higher incidence of LC and better OS than black males [43].

The study by Ding et al. analyzed the effect of marital status on OS and DSS in patients with LC. Marriage appears to have a more significant protective impact on women than on men, improving their OS (59% vs. 58.50%) and DSS (70.80% vs. 68.60%) [49]. This may reflect a greater propensity of women to seek medical care and adhere to treatments. These aspects highlight the importance of considering social and psychological factors in the treatment and management of patients with LC, with a particular focus on the specific needs of women.

These findings underscore the complexity of LC survival rates and highlight the need for further research into factors influencing survival, including race, age, and healthcare disparities.

## 7. Psychological Impact and Fear of Recurrence (FoR)

Women with LC tend to experience a greater psychological impact than men, leading to a negative impact on their quality of life and in some cases even survival. Indeed, many studies reported that women affected by head and neck cancer appear particularly vulnerable to negative psychological effects related to gender expectations and social norms. In particular, women tend to be more affected by concerns about their physical appearance, the quality of their voice, their role in caring for others, and social integration. As a result, these challenges can lead to higher rates of anxiety and depression than those of men. It was also stressed that there are specific needs for gender, especially in terms of speech rehabilitation and social reintegration, which require a targeted approach to ensure optimal recovery and improvement of quality of life [50,51,52,53,54,55].

Awareness of the professional etiology for the development of LC also has its psychological impact on patients. Indeed, Bonafede et al. reported that the occupational risk of LC is not only a medical issue but also a psychological one, with different implications for men and women. Women exposed to occupational risks related to LC (i.e., exposure to asbestos, industrial dust, or chemicals) appear to be more sensitive to the emotional implications of these risks. This may be due to a more acute perception of the potential impact on their health and quality of life [56].

**Figure 2:**
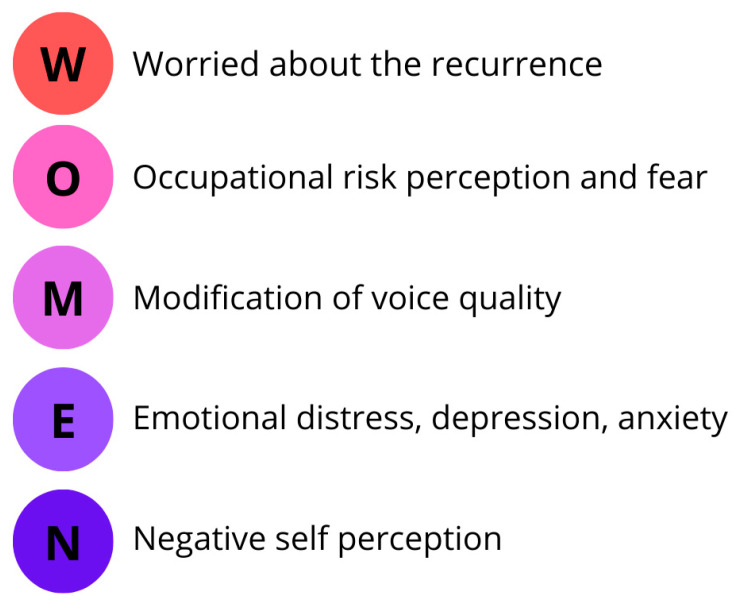
Psychological impact of laryngeal cancer on women.

**Figure 3:**
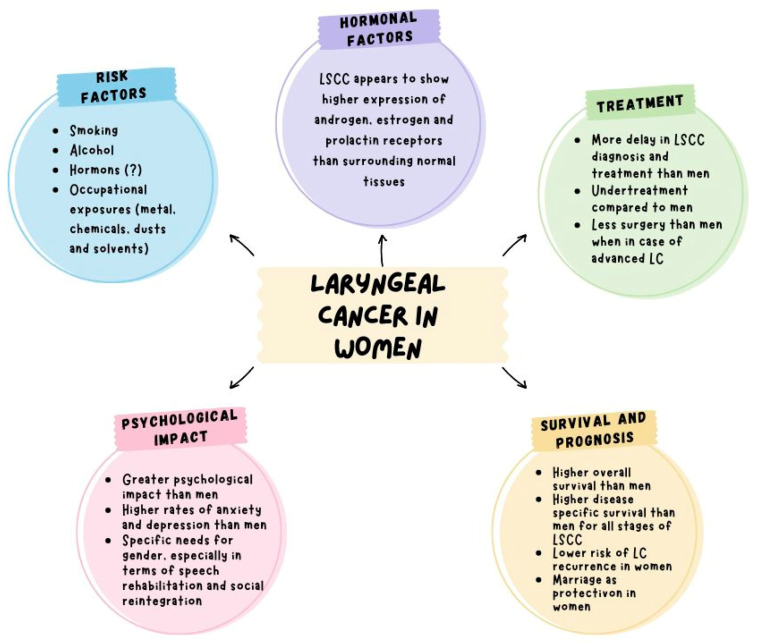
Summary of characteristics of laryngeal carcinoma in women.

The fear and worry of cancer relapse are a significant physical and mental burden for many patients and their partners, leading to emotional distress, depression, anxiety, and worse quality of life. The study by Muldbücker et al. compared the levels of FoR between patients with LC and their partners without finding a significative difference (*p*-value 0.029). Moreover, they found higher levels of FoR in female patients (mean = 35.76) than in female partners (mean 27.11) [57]. This increased anxiety may be linked to the emotional and psychological challenges faced by women battling cancer. Addressing the mental health aspects of cancer care is critical to improve quality of life for female patients (Figure 2 and Figure 3, Table 1).

## 8. New Perspectives in Research

One of the most worrying aspects of the research analysis is the lack of consideration of gender variables in clinical investigations. Although the importance of biological differences between men and women is well documented, only a small percentage of studies take these factors into account, with only 35% of studies reporting sex-disaggregated data and only 10% analyzing gender variables [58]. This research gap could limit understanding of differences in cancer biology and therapeutic responses, hampering progress in the personalization of treatments for women. Biological sex, which affects the physiological differences between men and women, can affect how a cancer develops and responds to drugs. Women and men may also react differently to treatments, not only for biological reasons but also because of gender-related psychological and social factors. Future research needs to fully integrate gender variables, not only to improve the understanding of laryngeal cancer, but also to optimize prevention, diagnosis, and treatment strategies.

## 9. Discussion

LC in women is a complex issue, which has received less attention in the scientific literature compared to LC in men, despite the increased relevance of this type of cancer in females as well. As shown, the disparity in incidence between men and women is considerable, with an M:F ratio of approximately 4:1 [4], with women continuing to be significantly less affected. However, a closer analysis of the temporal trend suggests an overall decrease in LC cases, which was more marked among men than women [9]. If this trend continues, it is possible that the incidence of LC among women will see a further increase, complicating the overall picture of the disease. It is important to note that the incidence of LC among women is not homogeneous: it varies significantly by region and population, which could reflect both genetic predispositions and cultural differences in lifestyle and access to health care. For instance, countries like Cuba and Hungary have significantly higher LC rates in women, while regions such as Benin and Guadeloupe report lower rates. This geographical variability requires more targeted research into the socio-economic, environmental, and lifestyle factors that could explain these differences.

The risk factors for LC in women appear to be similar to those in men, with smoking and alcohol consumption as the main causes of disease development [16]. However, the literature suggests that the risk associated with tobacco consumption may be higher in women than in men, although alcohol, while being a risk factor, plays a less significant role than smoking, except in cases of excessive consumption [17,18]. Interestingly, women may be more susceptible to the effects of smoking and alcohol, not only due to behavioral factors but also due to biological factors related to different hormonal responses between the sexes. Sex hormones, especially estrogen and androgen, seem to play a key role in determining laryngeal susceptibility to tumor development [33,34,35,36]. The interaction between these hormones and specific receptors in cancer cells can accelerate tumor growth and complicate treatment, making the understanding of hormonal mechanisms critical to improving therapeutic strategies. The relationship between hormonal factors and LC is particularly interesting in menopausal women, as the loss of estrogen can alter the microenvironment of the tumor and increase susceptibility to more aggressive forms of cancer. Research suggests that early menopause, particularly before age 52, may be associated with a higher risk of head and neck cancer, including LC.

Another noteworthy aspect is the role of occupational exposures in increasing LC risk. Some occupations, such as blacksmith work and work with electrical or electronic equipment, have been found to be a potential risk factor, particularly for women. Occupational exposures such as to chemicals, dusts, and solvents may disproportionately affect women, requiring more attention in assessment of health risks at work and in defining protective measures.

Another interesting aspect is the survival of women with LC. Several studies have documented higher OS in women than in men, particularly for glottic and supraglottic cancers. This finding may be related to the fact that women, on average, have a lower exposure to smoking and a higher propensity to quit smoking after diagnosis, factors which positively affect prognosis [4,46]. However, studies showed differences in incidence and survival between males and females, according to the state in which the study was conducted [4,20]. It is therefore essential to investigate how social and health variables can influence survival to optimize the care of women with LC.

Regarding treatments, the analysis of therapeutic protocols revealed differences in treatment between men and women, with women appearing to be treated less intensively. For example, it has been found that women are less likely to receive combinations of cisplatin and radiation therapy than men. They also tend to be treated more frequently with radiotherapy than surgery, especially in cases of advanced disease [40]. These differences could be due to gender-related factors, such as the perception of disease, age, or response to treatments, and they suggest the need for more personalized therapeutic approaches, taking account of the specific characteristics of each sex.

In addition to biological and clinical factors, it is important to consider socio-economic and psychological aspects when examining gender disparities in the outcome of LC. Studies have shown that women with CL experience higher levels of anxiety and depression than men, probably due to social expectations about their physical appearance, their roles as caregivers, and the stigma associated with head and neck cancer. This psychological impact could affect therapeutic choices, further complicating the clinical management of LC in women. Moreover, studies reported that women experience increased emotional distress related to their physical appearance and voice, which are often altered due to LC treatments. These fears may affect their social reintegration, rehabilitation, and quality of life after treatment. Addressing these psychological and emotional needs is crucial to ensure the best healthcare and support and the best quality of life after LC diagnosis and treatment.

## 10. Conclusions

Although LC in women has historically been little studied compared to that in men, the available evidence shows significant gender differences in incidence, disease progression, survival, and treatment. These differences suggest that women may have a different response to laryngeal cancer than men, influenced by biological, hormonal, and social factors which need to be better understood. For example, the hormonal mechanisms which can influence the development and progression of laryngeal carcinoma deserve more attention, as do environmental factors which could have a different impact on men and women. In addition, disparities in treatment, with women often receiving less aggressive care than men, indicate the need to address these differences in therapeutic protocols, to ensure that patients receive the most appropriate and personalized treatment. In the future, it is essential that research integrates gender variables not only to optimize prevention and diagnosis strategies but also to develop more targeted treatments which meet the specific needs of patients. Biological differences between the sexes, for example, could influence the response to treatments and consequently the effectiveness of therapies. In addition, psychological and socio-cultural factors such as the role of women in society and their expectations regarding quality of life after treatment should be included in therapeutic decisions, to ensure that care is truly personalized.

The increasing incidence of LC among women underlines the need to continue efforts to research and improve the quality of care. It is crucial to ensure that women receive the best possible treatment and that the best clinical outcomes are achieved, both in terms of survival and quality of life. Only a more inclusive and gender-sensitive approach will improve outcomes for patients with LC, ensuring that their specific therapeutic and psychological needs are not overlooked.

## Figures and Tables

**Table 1:** Differences between men and women affected by laryngeal carcinoma.

Features	Men	Women
*Age-standardized incidence rate in the world per 100,000 persons* [7]	Cuba (14.8) Romania (10.3) Georgia (9.8)	Cuba (2.3) Hungary (1.3) Syrian Arab Republic (1.2)
*Peak incidence of LC* ^1^	65 years old	
*(cases per 100,000 persons)* [3,7,11,12]	25–30	4
*LC* ^1^ *prevalence*	Between 60 and 80 years old	
*(cases per 100,000 persons)* [7]	120–140	17–20
*Mortality peak due to LC* ^1^	At around 80 years old	
*(cases per 100,000 persons)* [3,7,11,12]	23	3
*Risk factors* [15,16,17,18,19,20,21,22,23,24,25,26,27]	Smoking and alcohol Occupational exposures	Smoking Alcohol (less important than smoking) Occupational exposures
*Laryngeal site involved by LC* ^1^		
Glottis	42.4% [20]|64.2% [4]	26.4%
Supraglottis	14.2%	24.4%|61.2% [4]
*Hormonal factors promoting LC* ^1^ [28,29,30,31,32,33,34]		Placental growth factor (pregnancy), androgen, estrogen, and prolactin receptors
*Treatment (first choice)* [4,38]		
Surgery	48.2%	48.2%
Conservative therapy	45.3%	56.8%
*Prognosis* [43,44,45,46,47]		
*OS* ^2^	Worse	Better
*DSS* ^3^	Worse	Better
*LC 1 recurrence*	Higher	Lower
*Anxiety, depression, emotional distress, worse quality of life* [50,51,52,53,54,55,56]	Less	More

^1^ LC: laryngeal cancer; ^2^ OS: overall survival; ^3^ DSS: disease-specific survival.
